# Sulphur in Typhoid

**Published:** 1915-11

**Authors:** 


**Sulphur in Typhoid.** Goubeau, L'Union Pharmaceutique, reports 31 cases without a death, following the suggestion of Vorochilsky and Burzagli. An initial calomel purge is given, then washed sublimed sulphur in doses of 5-6 grams, 5-6 times a day. A general mitigation of symptoms and shortening of course is noted. (Note: Acetozone, salacetol with animal charcoal, various other internal antiseptics, colonic lavage, etc., have likewise given good results. While typhoid has been shown to be a general infection, almost any method, even including diet that produces a fair approach to intestinal asepsis, certainly reduces mortality. It may almost be said that typhoid without modification of the intestinal flora, is typhoid plus mixed infection or, at least, typhoid plus intoxication from other bacterial toxins.

